# Supplementary material for: Characterization of a KDM5 small molecule inhibitor with antiviral activity against hepatitis B virus
Source: PLoS One. 2022 Dec 7;17(12):e0271145. doi: 10.1371/journal.pone.0271145 (PMC9728921; doi:10.1371/journal.pone.0271145)
Supplement: S1 File — (DOCX) [file pone.0271145.s001.docx]

**Supporting Information**

**
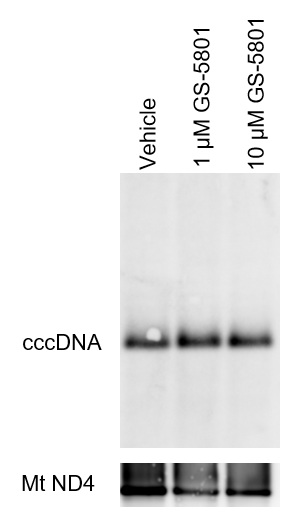
**

**S1 Fig. GS-5801 does not reduce levels of cccDNA after 14 days of treatment.** PHH were infected with HBV for three days prior to initiation of GS-5801 treatment. PHH were dosed with vehicle, 1 µM GS-5801, or 10 µM GS-5801 every three to four days for a total of 14 days. Total DNA was isolated, treated with T5 exonuclease to reduce levels of relaxed circular HBV DNA (rcDNA), and probed for cccDNA and mitochondrial DNA (Mt ND4: NADH-ubiquinone oxidoreductase chain 4) by Southern blot.

**
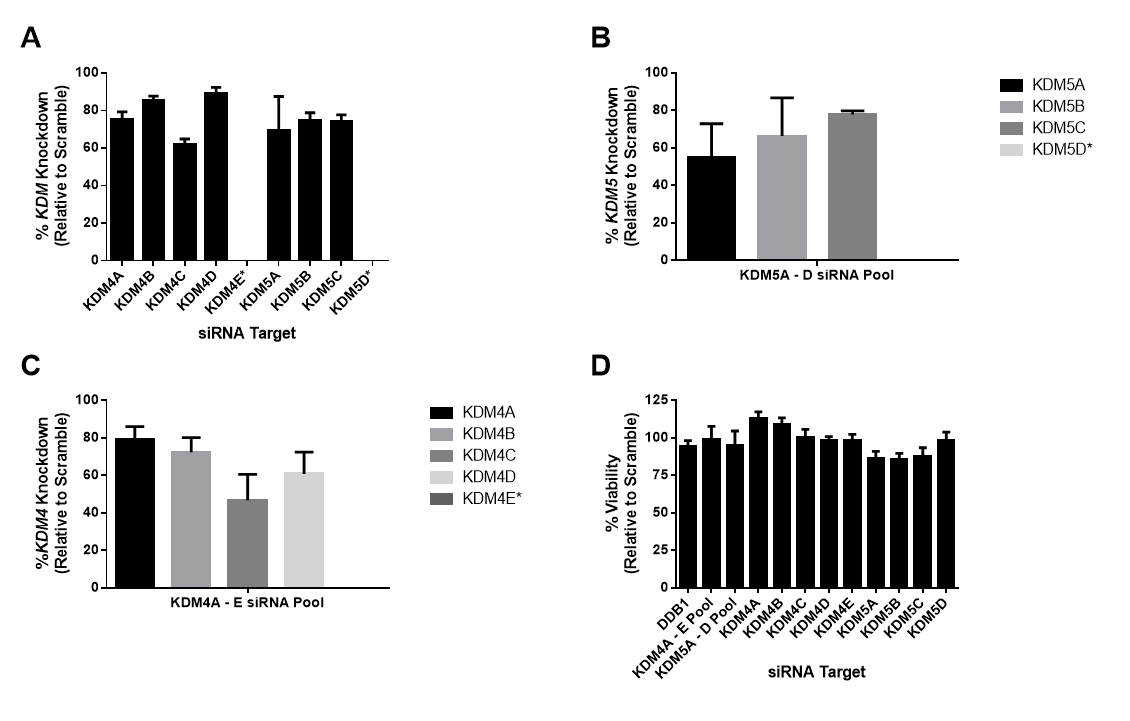
**

**S2 Fig. siRNA knockdown of *KDM* transcripts in PHH.** *KDM4* and *KDM5* transcripts were depleted by siRNA either individually (*KDM4A*, *B*, *C*, *D*, or *E*; *KDM5A*, *B*, *C*, or *D*) or simultaneously (*KDM4A* – *E* pool; *KDM5A* – *D* pool) in HBV-infected PHH. The percentage knockdown of each *KDM* transcript relative to a scramble siRNA control is shown for (A) *KDM4* and *KDM5* transcripts depleted individually, (B) *KDM5A* – *D* transcripts depleted simultaneously (*KDM5A* – *D* pool), and (C) *KDM4A* – *E* transcripts depleted simultaneously (*KDM4A* – *E* pool). (D) The viability of HBV-infected PHH on Day 14 after initiation of siRNA treatment was assessed by alamarBlue staining. PHH viability is shown as the percentage viability relative to a scramble siRNA control. Data shown are the average of two biological replicate experiments and error bars represent the standard deviation. *, expression of transcript was not detected by qRT-PCR in PHH and thus could not be assessed for percent knockdown by siRNA.

**
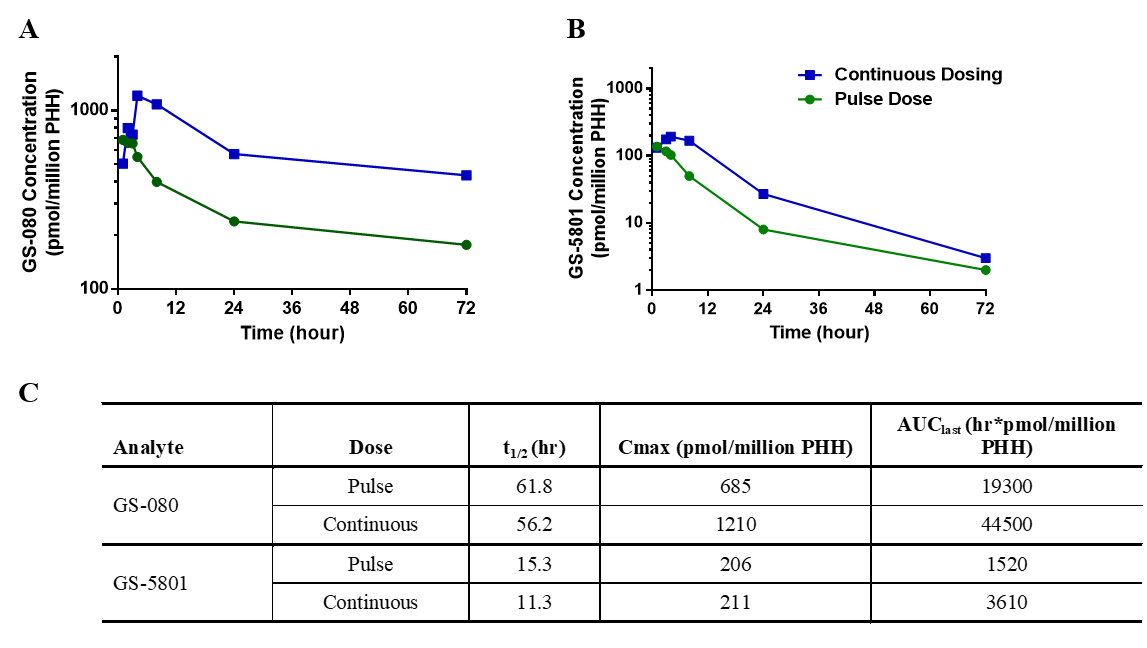
**

**S3 Fig. GS-5801 pulse dose reduces cell-associated GS-080 exposure.** PHH were infected with HBV for three days prior to initiation of GS-5801 treatment. PHH were dosed with vehicle or 1 µM GS-5801once for three days (three day exposure; continuous dosing) or once for two hours (two hour exposure; pulse dosing). PHH were harvested at 1, 2, 3, 4, 8, 24, and 72 hours after GS-5801 treatment with pulse or continuous dosing and levels of (A) GS-080 active parent or (B) GS-5801 pro-drug were measured by liquid chromatography followed by mass spectrometry (LC-MS) at each timepoint. (C) Pharmacokinetic parameters calculated included the half-life (t_1/2_), maximum concentration (C_max_), and area under the curve (AUC_last_).

**
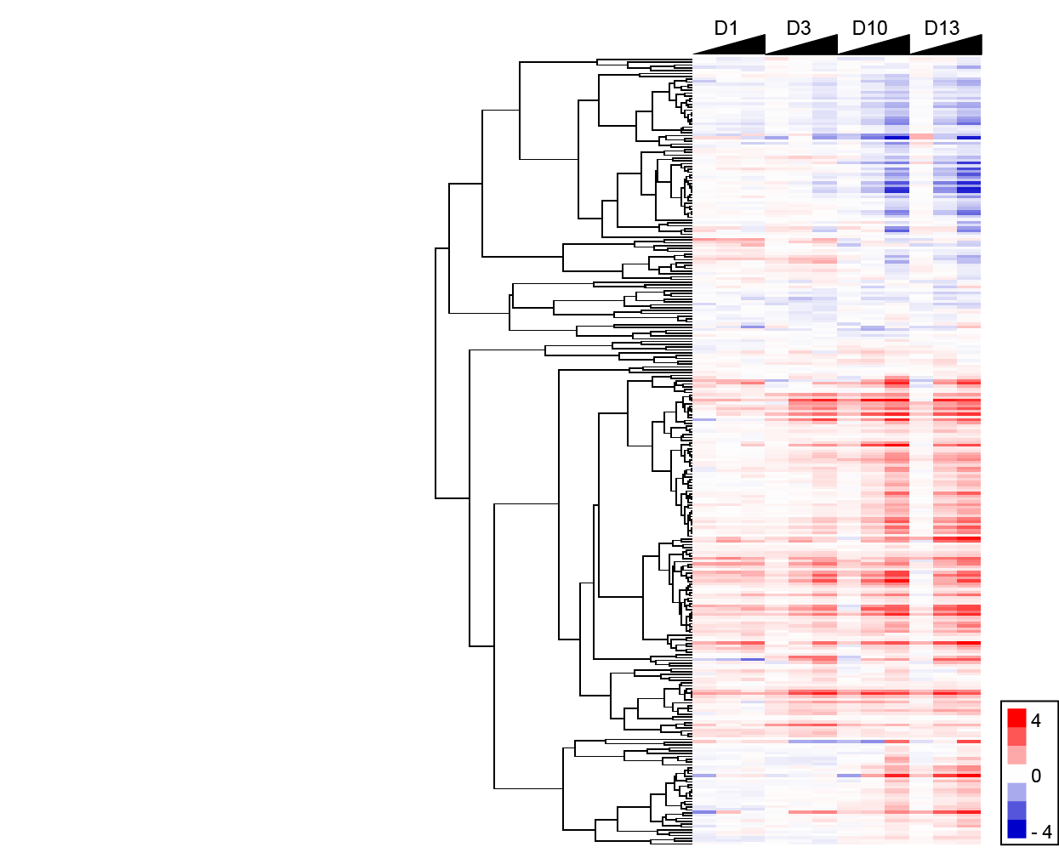
**

**S4 Figure. GS-5801 impact on ISGs**. Interferon-stimulated genes (ISG) expressed in PHH (n = 279) were examined by hierarchical clustering using an uncentered Pearson correlation. Data are displayed as a heatmap of log2 differential cpm values with red representing genes upregulated in GS-5801 treated PHH and blue representing genes downregulated in GS-5801 treated PHH

**SI Table 1. GS-5801 and GS-080 do not exhibit cytotoxicity at doses up to 57 µM**

| Compound | Cytotoxicity CC_50_ (µM)^a^ | | | | | | | |
| --- | --- | --- | --- | --- | --- | --- | --- | --- |
|  | MT-4^b^ | PBMC | Gal-HepG2 | Huh-7 | PHH | Gal-PC3 | MRC-5 | NRVM |
| GS-5801 | 51  ± 1.2 | > 44 | > 44 | > 44 | > 50 | > 44 | > 44 | > 44 |
| GS-080 | > 57 | > 44 | > 44 | > 44 | > 50 | > 44 | > 44 | > 44 |
| Puromycin | 0.10  ± 1.3 | 0.61  ± 1.3 | 1.3  ± 1.4 | 0.67  ± 1.5 | 1.6  ± 1.8 | 0.44  ± 1.5 | 0.35  ± 1.4 | 0.57  ± 1.4 |

a Values represent the geometric mean and multiplicative standard deviation of 3 – 5 primary human hepatocyte (PHH) donors or 5 – 10 independent experiments.

b MT-4: human T-lymphoblastoid cell line; PBMC: peripheral blood mononuclear cell; Gal-HepG2: galactose adapted human hepatoblastoma cell line; Huh-7: human hepatoma cell line; Gal-PC3: galactose adapted human prostate carcinoma cell line; MRC-5: Medical Research Council 5 embryonal lung fibroblast; NRVM: neonatal rat ventricular cardiomyocytes

**SI Table 2. GS-718420, a brominated derivative of GS-5801, does not exhibit antiviral activity in HBV-infected PHH**

| PHH Donors^a^ | Hu8181 | Hu8130 | Hu4167 | BCD | Hu7272 | Hu276 | Hu349 |
| --- | --- | --- | --- | --- | --- | --- | --- |
| vRNA  EC_50_ (μM) | > 10 | > 10 | > 10 | > 10 | > 10 | > 10 | > 10 |
| vDNA  EC_50_ (μM) | > 10 | > 10 | > 10 | > 10 | > 10 | > 10 | > 10 |
| HBsAg  EC_50_ (μM) | > 10 | > 10 | > 10 | > 10 | > 10 | > 10 | > 10 |
| HBeAg  EC_50_ (μM) | > 10 | > 10 | > 10 | > 10 | > 10 | > 10 | > 10 |
| CC_50_ (μM) | > 10 | > 10 | > 10 | > 10 | > 10 | > 10 | > 10 |

a Values represent the average EC_50_ values for GS-718420 from n = 1 donor Hu8181, n = 2 donor Hu8130, n = 2 donor Hu4167, n = 1 donor BCD, n = 1 donor Hu7272, n = 2 donor Hu276, n = 2 donor Hu349 experiments.

**SI Table 3. Biochemical potency of GS-080 against histone methyltransferase and histone deacetylase enzymes**

| Enzyme Family | Enzymes | Enzyme Conc. (nM) | Positive Controls  IC_50_ (µM)^a^ | IC_50_ (µM) |
| --- | --- | --- | --- | --- |
| Histone methyltransferase | DOT1L | 2 | SAH (0.480) | > 100 |
|  | NSD1 | 50 | SAH (93) | > 100 |
|  | NSD2 | 10 | SAH (11) | > 100 |
|  | NSD3 | 100 | SAH (15) | > 100 |
|  | G9a | 100 | SAH (7.5) | > 100 |
|  | SET7/9 | 100 | SAH (70) | > 100 |
|  | GLP | 100 | SAH (2.2) | > 100 |
|  | SETDB1 | 100 | SAH (17) | > 100 |
|  | PRMT1 | 100 | SAH (1.6) | > 100 |
|  | PRMT4 | 100 | SAH (0.43) | > 100 |
|  | PRMT5/MEP50 | 100 | SAH (1.5) | > 100 |
|  | EZH2/EED/SUZ12 | 100 | SAH (64) | > 100 |
| Histone deacetylase | HDAC-1 | 5 | Trichostatin A (0.0068) | > 100 |
|  | SIRT-1 | 5 | EX-527 (35) | > 100 |

a The IC_50_ values of positive control compounds are shown in parentheses.

**SI Table 4**. **Human KDM enzyme sources, protein constructs, expression systems, and substrates**

| KDM Enzyme | Source^a^ | Protein Construct Amino Acids | Expression Organism  and Tags | Substrate Peptide | Substrate  Peptide Conc. (nM) |
| --- | --- | --- | --- | --- | --- |
| 5A | BPS | 1-1090 | Bac, FLAG-tag C-term | H3(1-19)K4-me3 | 25 |
| 5B | RBC | 2-752 | Bac^b^, FLAG/His N-term | H3(1-21)K4-me3 | 30 |
| 5C | BPS | 2-1560 | Bac, FLAG/His N-term | H3(1-19)K4-me3 | 100 |
| 5D | BPS | Full length | Bac, FLAG/His N-term | H3(1-21)K4-me3 | 100 |
| 4A | BPS | 1-350 | *E. coli*, His-tag N-term | H3(1-19)K9-me3 | 100 |
| 4B | BPS | 2-500 | Bac, GST-tag N-term | H3(1-19)K9-me3 | 100 |
| 4C | BRIC | 1-349 | *E. coli*, His-tag N-term | H3(1-19)K9-me3 | 200 |
| 1A | BPS | 158-852 | *E. coli*, GST-tag N-term | H3(1-21)K4m1 | 80 |
| 2A | BPS | 2-700 | Bac, FLAG-Avi-tag N-term | H3(26-47)K36-me2 | 50 |
| 2B | BPS | 1-650 | Bac, FLAG-tag C-term | H3(26-47)K36-me2 | 50 |
| 3A | BPS | 2-1322 | Bac, FLAG-tag N-term | H3(1-19)K9-m2 | 50 |
| 3B | BRIC | 842-1761 | Bac, His-tag N-term | H3(1-19)K9-m2 | 100 |
| 6A | BRIC | 919-1401 | *E. coli*, His-tag N-term | H3(21-44)K27-m3 | - |
| 6B | BPS | 1043-1643 | Bac, FLAG-tag C-term | H3(21-41)K27-m3 | 100 |
| 7B | BRIC | Full length | Bac, FLAG/His N-term | H3(1-19)K9-m2 | 100 |

a Abbreviations. BRIC: Dr. Jesper Christensen at Biotech Research & Innovation Centre at the University of Copenhagen; BPS: BPS Biosciences (San Diego, CA); RBC: Reaction Biology Corporation (Malvern, PA).

b Bac = Baculovirus-infected Sf9 cell expression system.

SI Table 5. Histone methyltransferase/demethylase sources, protein constructs, and substrates

| Enzyme | Sources^a^ | Protein Construct Amino Acids | Protein or Peptide Substrates |
| --- | --- | --- | --- |
| DOT1L | BRIC | 1-416 | Bovine thymus nucleosome antigen |
| NSD1 | RBC | 1538-2696 | HeLa cell nucleosomes |
| NSD2 | RBC | Full Length | HeLa cell nucleosomes |
| NSD3 | RBC | 1021-1322 | HeLa cell nucleosomes |
| G9a | RBC | 786-1210 | Chicken core histones |
| SET7/9 | RBC | 2-366 | Chicken core histones |
| GLP | RBC | 894-1298 | Chicken core histones |
| SETDB1 | BPS | 510-1290 | Chicken core histones |
| PRMT1 | BPS | 2-371 | Chicken core histones |
| PRMT4 | BPS | 2-608 | Chicken core histones |
| PRMT5/MEP50 | BPS | 2-637/3-342 | Chicken core histones |
| EZH2/EED/SUZ12 | BPS | 2-746/2-441/2-739 | Chicken core histones |
| HDAC-1 | BPS | Full length | Fluorogenic peptide from p53 residues 379-382 [RHKK(Ac)AMC] |
| SIRT-1 (Sirtuin 1) | BPS | 193-741 | H3(1-21)K4Ac |

a Abbreviations. BRIC: Dr. Jesper Christensen at Biotech Research & Innovation Centre at the University of Copenhagen; BPS: BPS Biosciences (San Diego, CA); RBC: Reaction Biology Corporation (Malvern, PA)

SI Table 6. RNAseq data from PHH treated with GS-5801

Separate file showing the fold change in transcript expression levels of PHH treated with GS‑5801 (0.03 µM, 0.3 µM, and 10 µM) compared to vehicle treated PHH on Day 1, Day 3, Day 10, and Day 13. Shown are log_2_ fold change values as well as false discovery rate values for each transcript measured.
